# Validation and quality assurance for genome browser database exports

**DOI:** 10.1186/1471-2105-16-S15-P13

**Published:** 2015-10-23

**Authors:** Roger Chui, Jerzy W Jaromczyk, Neil Moore, Christopher L Schardl

**Affiliations:** 1 Department of Computer Science, University of Kentucky, Lexington, KY 40506, USA; 2 Department of Plant Pathology, University of Kentucky, Lexington, KY 40546, USA

## Background

A genome browser transition utility designed in our lab, FPD2GB2 (Fungal Project Database to GBrowse 2), exports data from a custom database used by the Fungal Endophytes Genome Project[1,2]. Designed as a collection of scripts, FPD2GB2 outputs the contents of a locally developed genome annotation database into the standard GFF3 format, allowing for bulk import of data into the GBrowse2 genome browser[3]. In short, FPD2GB2 is a collection of scripts designed to export data encoded in the Fungal Project Database format into a format which can be easily imported into GBrowse 2, namely GFF3.

## Materials and methods

Any application which converts between data formats should ensure the completeness and accuracy of the output produced by FPD2GB2. Adding a data validator as part of the FPD2GB2 script collection allows for independent verification of the quality and soundness of the GFF3 files being imported into a production GBrowse2 environment.

**Figure 1:**
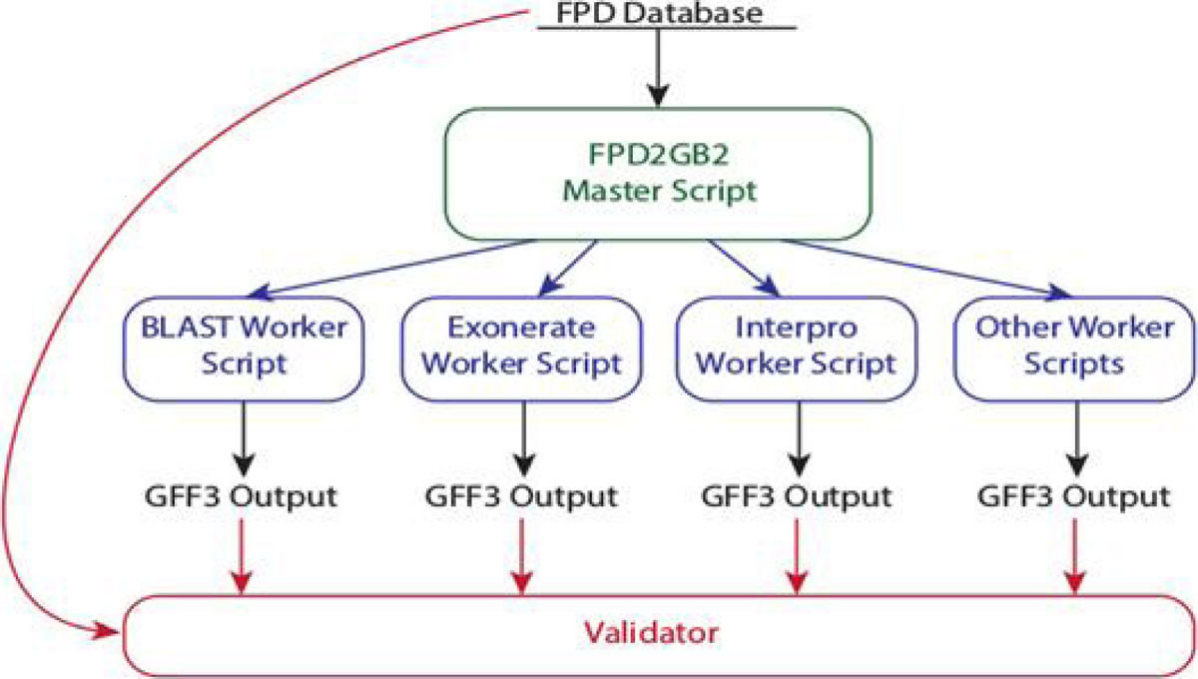
Block diagram of FPD2GB2 data flow and execution.

We measure the accuracy of the output by comparing the features listed in the GFF3 files to the contents of the original database. Ensuring accurate offsets relative to reference features provides validation of accuracy. Comparing the parent-child inheritance structure of features in the output to that of the source data ensures the completeness of the output. The script collection is structured into a “master” script and several “worker” scripts, each of which produces its own output. The structure of the collection is shown in Figure 1. The goals and methods for the validator are described in Table 1.

**Table 1:** Goals and methods for the validator.

*Completeness:* Verify that all data has been exported.	Count base features in original database, ensure the count of features in output is an exact match.
***Correctness:* Ensure that the content exported accurately represents the original copy.**	Manual checking of a subset of features is a pragmatic way to ensure correctness, at least initially.

***Accuracy:* Check the placement of annotations relative to references to ensure there are no off-by-one errors.**	Select a set of features to use as reference. Calculate distance to other features in both FPD database and GFF3 file. Make sure that features in tracks that are directly related (e.g. exonerate results are derived from BLAST results) correspond at a high rate using a feature comparator such as ParsEval (part of the genometools package which is available at http://genometools.org/).

***Standards Compliance:* Verify output conforms to GFF3 standard http://www.sequenceontology.org/gff3.shtml**	Use gff3validator (also part of the genometools package) to ensure compliance with the GFF3 standard.

## Results

It is notoriously difficult to prove accuracy of computational results and in practice validation is based on testing. In our case to validate the completeness, correctness and accuracy we use metrics which can not only give confidence that the output tends to accurately reflect the output, but also that the algorithms used to create the output are correct. The size of some of the databases and number of annotation tracks also makes full comparison of related tracks impractical, as fully comparing tracks takes a quadratic number of runs with respect to the number of tracks. Finally, because of the way the annotations do not have metadata establishing relationships, comparisons using ParsEval have to be run manually.
